# Metabolic syndrome in young Asian Indian patients with myocardial infarction

**Published:** 2007

**Authors:** N Ranjith, RJ Pegoraro, DP Naidoo, TM Esterhuizen

**Affiliations:** Department of Medicine, Coronary Care Unit, RK Khan Hospital, Durban; Department of Chemical Pathology, Nelson R Mandela School of Medicine, University of KwaZulu-Natal, Durban; Department of Cardiology, Inkosi Albert Luthuli Central Hospital, Durban; Medical Bioethics Unit, University of KwaZulu-Natal, Durban

## Abstract

**Objectives:**

This study assessed the prevalence of the metabolic syndrome and its impact on hospital outcomes in young South African Indians (≤ 45 years) with acute myocardial infarction (AMI) using both the National Cholesterol Education Programme Adult Treatment Panel III (NCEP ATP III) and the International Diabetes Federation (IDF) definitions.

**Methods and results:**

The study population comprised 389 patients with AMI. The metabolic syndrome as defined by the NCEP ATP III criteria was found in 235 (60%) patients and in 223 (57%) according to the IDF criteria, with only a 79% concordance between the two definitions. However, when ethnic-specific waist circumference cut-offs proposed by the IDF were used as a criterion for obesity in the NCEP ATP III definition, the number of patients with the metabolic syndrome increased significantly to 270 (69%) (*p* < 0.001).

Elevated fasting blood glucose was the major NCEP ATP III determinant present in 86% of individuals. All determinants for both definitions were found more frequently in patients with the metabolic syndrome (*p* < 0.001). Although 44% of patients had triple-vessel disease on cardiac catheterisation studies, the frequency of adverse cardiovascular events during hospital stay was low, and was uninfluenced by the presence or absence of the metabolic syndrome.

**Conclusion:**

The metabolic syndrome is a common finding in young Indian patients with AMI who frequently present with extensive atherosclerotic disease. Adverse event rate during hospital stay was low, and was unrelated to the presence of the metabolic syndrome. There was no significant difference in the prevalence rate of the metabolic syndrome as determined by either the NCEP ATP III or IDF definitions, but there was only a moderate level of agreement between the two definitions. Inclusion of ethnic-specific waist circumference cut-offs as the determinant of obesity in the NCEP definition may identify more accurately individuals at increased cardiometabolic risk and improve predication of the metabolic syndrome.

## Summary

Asian Indians have a high incidence of coronary heart disease (CHD), while Asian Indian immigrants throughout the world have been shown to have an increased CHD mortality rate compared with the native populations of their adopted countries. 1 In South Africa, CHD in the Asian Indian population has reached similar epidemic proportions.^2^ This group also has an increased incidence of premature atherosclerotic disease; approximately 20% of patients with an AMI are 45 years of age or younger.^3^ By way of comparison, both in Mediterranean patients and in a study undertaken in the United States, the reported incidence of myocardial infarction (MI) in patients of similar age was between 4 and 10%.^4^,^5^ In addition, many young South African Indian patients have been found to have several components of the metabolic syndrome such as dyslipidaemia, characterised by elevated triglycerides (TG) with a reduction in high-density lipoprotein (HDL) cholesterol concentrations, and obesity.^3^

It is now well recognised that the prevalence of the metabolic syndrome varies among different ethnic groups.^6^ In comparison with European subjects, South Asians have higher mean waist−hip ratios, contributing to their greater risk of developing insulin resistance, hyperinsulinaemia, hypertension, hypertriglyceridaemia and diabetes.^7^ The predominance of visceral adipose tissue with increasing waist circumference in Asian Indians^8^ renders the prevalence of the metabolic syndrome higher in this group than in African−Americans in whom subcutaneous fat predominates.^9^ Furthermore, the prevalence of the metabolic syndrome is highly age dependent, with the reported incidence being less than 10% in individuals in the 20- to 29-year age group and increasing to between 38 and 67% in the 60- to 69-year age group.^10^

In addition to the effects of age and ethnicity, the diagnostic criteria used to define the metabolic syndrome further influence the prevalence rates. One definition used is the NCEP ATP III,^11^ which facilitates a clinical diagnosis of high-risk individuals for cardiovascular disease. A particular problem with the NCEP ATP III definition has been its applicability to different ethnic groups, especially as relates to obesity cut-offs. Using this definition results in lower prevalence figures for the metabolic syndrome in Asian populations.^12^ This highlights the need for lower cut-off values for waist circumference in populations known to be at high risk for insulin resistance-mediated cardiometabolic disease. More recently, the IDF has proposed a new definition, with the aim of establishing global consensus on a unified working diagnosis that can be used universally to allow comparison of data.^13^ This definition incorporates ethnic-specific waist circumference cut-offs, based on data linking waist circumference to other components of the metabolic syndrome in different populations.^14^,^15^

Irrespective of the type of definition employed, many studies have shown the metabolic syndrome to be associated with an increased risk of cardiovascular disease.^16^-^18^ The metabolic abnormalities common to this syndrome, such as insulin resistance, central obesity, dyslipidaemia and hypertension are all independent risk factors for the development of cardiovascular disease, which, when grouped together, amplify this risk. There are limited data on the risks associated with the metabolic syndrome in the increasingly large group of young patients who have sustained an AMI.

The aim of this study, therefore, was to assess and compare the prevalence of the metabolic syndrome and its impact on hospital outcomes in young South African Indian patients with AMI, by using both the NCEP ATP III and the IDF definitions.

## Methods

The study population was recruited from consecutive patients admitted with a diagnosis of AMI to the Coronary Care Unit (CCU) at RK Khan Hospital, Durban, over a five-year period (May 1999 to June 2005). A total of 389 patients who were 45 years and younger was available for analysis, all of whom were of Asian Indian origin. Informed consent was obtained prior to any study-related procedures. Acute myocardial infarction was defined as at least two of the following: prolonged chest discomfort, typical electrocardiographic changes, or elevated cardiac troponin levels, as outlined by the Joint European Society of Cardiology/American College of Cardiology Committee.^19^

Demographic data were obtained from all patients. These included age, gender, weight, height, waist circumference, and information on risk factors such as diabetes, hypertension, smoking status, and a family history of vascular disease. Waist circumference was measured with a soft tape measure, on standing subjects, midway between the lowest rib and the iliac crest.

Additional clinical data on complications encountered from hospital admission until discharge were collected, and included events such as sustained ventricular arrhythmias (ventricular tachycardia/ventricular fibrillation) requiring intervention, complete heart block, cardiac failure, cardiogenic shock, recurrence of angina or MI and death, as well as information on angiographic results and revascularisation procedures such as angioplasty and coronary artery bypass grafting (CABG). Cardiac failure was defined as progressive resting dyspnoea associated with clinical signs of pulmonary and/or peripheral congestion, based on Framingham criteria requiring hospitalisation and treatment with diuretics. Coronary anatomy was described using the criteria of the Coronary Artery Surgery Study (CASS).^20^

The presence of the metabolic syndrome as outlined by the NCEP APTIII criteria was defined as three or more of the five risk factors shown in Table 1. The IDF used lower cut-offs for waist measurement in Asian Indians, as well as lower fasting plasma glucose levels, and defined the metabolic syndrome as central obesity (waist circumference: males ≥ 90 cm, females ≥ 80 cm) plus any two of the criteria shown in Table 1.

**Table 1:** Prevalence Of The Metabolic Syndrome According To The NCEP ATP III And IDF Definitions And The Frequency Of The Individual Criteria

	*Metabolic syndrome n = 235 (60%)*	
*NCEP criteria*	*Yes (n 5 235)*	*No (n 5 154)*
Fasting glucose ≥ 6.1 mmol/l*	202 (86%)	79 (51%)
TG ≥ 1.7 mmol/l*	194 (83%)	49 (32%)
HDL (males < 1.03) (females < 1.29) mmol/l*	173 (74%)	66 (43%)
Waist circumference (males > 102 cm)* (females > 88 cm)	142 (60%)	24 (16%)
Blood pressure ≥ 130/≥ 85 mmHg*	136 (58%)	25 (16%)
	*Metabolic syndrome n = 223 (57%)*	
*IDF criteria*	*Yes (n = 223)*	*No (n = 166)*
Waist circumference (males ≥ 90 cm)* (females ≥ 80 cm)	223 (100%)	41 (25%)
Fasting glucose ≥ 5.6 mmol/l*	202 (91%)	118 (71%)
TG > 1.7 mmol/l*	174 (78%)	69 (42%)
HDL (males < 1.03) (females < 1.29) mmol/l**	150 (67%)	89 (54%)
Blood pressure ≥ 130/≥ 85 mmHg*	113 (51%)	48 (29%)

In the IDF definition patients on specific treatment for blood pressure and fasting plasma glucose were included irrespective of the value obtained. *p < 0.001; **p < 0.05.

Troponin T levels were measured by a third-generation immunoassay method (Elecsys 1010, Roche Diagnostics), with the clinical discriminator value for MI being 0.1 ng/ml (detection range 0.010–25.00 ng/ml; sensitivity 100% and specificity 83.9% at 24 hours). Blood samples were also collected within 48 hours of admission after an overnight fast, for total cholesterol, triglycerides and HDL, which were measured on the Beckman Synchron CX7 auto analyser.

SPSS version 11.5 (SPSS Inc, Chicago, Ill, USA) was used for data analyses. Pearson’s chi-square test or Fisher’s exact test where appropriate were used to assess associations between categorical exposures and outcomes. The two-sample independent *t*-test was used to compare the means between two groups. The association between two independent categorical variables was assessed using Pearson’s chi-square test, while the McNemar’s chi-square test was used to compare paired proportions. Agreement between the two definitions of the metabolic syndrome was assessed using Cohen’s kappa statistic.

The ethics committee of the Faculty of Health Sciences, Nelson R Mandela School of Medicine, University of KwaZulu-Natal granted approval for the study.

## Results

The study group comprised 389 patients 45 years of age or younger with a diagnosis of AMI, 87% of whom were males. Twenty-two per cent of patients were being treated for hypertension while no one was on therapy for dyslipidaemia prior to the study entry.

## Prevalence and comparison of the metabolic syndrome

The metabolic syndrome, as defined by the NCEP ATP III criteria, was found in 235 (60%) of the study population and in 223 (57%) patients according to the IDF criteria. When the two definitions were applied concurrently, only 188 patients (48%) were diagnosed with the metabolic syndrome, while 119 (31%) were deemed not to have the syndrome, giving only a moderate agreement of 79% (kappa = 0.565) (Table 2). Of the remaining 82 (21%) patients, 35 (9%) had the metabolic syndrome based on the IDF criteria, but not the NCEP ATP III definition, while the remaining 47 patients (12%) were diagnosed with the metabolic syndrome according to the NCEP ATP III definition but not the IDF definition (McNemar’s chi-square, *p* = 0.224).

**Table 2:** Comparison Between NCEP ATP III And IDF Definitions Of The Metabol Ic Syndrome

		*Metabolic syndrome (IDF)*		**
		*Yes*	*No*	*Total*
Metabolic syndrome	Yes	188 (48%)	47 (12%)	235 (60%)
(NCEP ATP III)	No	35 (9%)	119 (31%)	154 (40%)
Total		223 (57%)	166 (43%)	389 (100%)

McNemar’s chi-square, *p* = 0.224 Cohen’s kappa = 0.565 (95% CI: 0.482−0.649).

Since the study population included only Asian Indian patients, it was decided to reassess the metabolic syndrome status using the waist criteria proposed by the IDF as a marker of obesity in the NCEP ATP III definition. This was based on the recommendations of the American Heart Association/National Heart, Lung and Blood Institute (AHA/NHLBI).^21^ Using the modified NCEP ATP III criteria resulted in 35 new cases being identified, significantly increasing the total number of patients diagnosed with the metabolic syndrome to 270 (69%) compared to 60% with the original NCEP ATP III definition (McNemar’s chi-square, *p* < 0.001). In addition, using the modified criteria, 48 more patients were diagnosed with the metabolic syndrome compared with the IDF definition, the difference being statistically significant (McNemar’s chi-square, *p* < 0.001).

As shown in Table 1, fasting blood glucose was the major NCEP ATP III determinant in patients with or without the metabolic syndrome (86% vs 51%), while raised triglyceride levels (83%), low HDL cholesterol levels (74%), central obesity (60%) and elevated blood pressure (58%) were all significantly more frequent in patients with the metabolic syndrome (Fisher’s exact, *p* < 0.001). Similar significant differences were observed in patients for the IDF definition of the metabolic syndrome, which required central obesity assessed by waist circumference as an essential component.

A family history of CHD, diabetes and hypertension was present in 61, 48 and 44% of patients, respectively. No significant differences existed in the frequency of a family history of vascular disease in patients with and without the metabolic syndrome, irrespective of the type of definition used.

## Adverse events during hospital admission

During hospitalisation, the most common adverse cardiovascular events seen were the recurrence of angina (9%) and cardiac failure (8%), while recurrent infarctions (3%), ventricular arrhythmias (3%) and complete heart block (1%) were observed less frequently (Table 3). No significant differences were observed for these event rates in patients with the metabolic syndrome compared to those without, for either of the definitions.

**Table 3:** Adverse Cardiovascular Events During Hospitalisation By The Metabolic Syndrome Definition

*Variables*	*Metabolic syndrome: NCEP ATP III*		*Metabolic syndrome: IDF*		*Metabolic syndrome: modified NCEP ATP III*	
	*Yes (n = 235)*	*No (n = 154)*	*Yes (n = 223)*	*No (n = 166)*	*Yes (n = 270)*	*No (n = 119)*
Recurrence of angina *n* = 34 (9%)	19 (8.1%)	15 (9.7%)	18 (8.1%)	16 (9.6%)	22 (8.1%)	12 (10.1%)
Cardiac failure *n* = 32 (8%)	19 (8.1%)	13 (8.4%)	17 (7.6%)	15 (9%)	20 (7.4%)	12 (10.1%)
Recurrence of infarct *n* = 12 (3%)	8 (3.4%)	4 (2.6%)	7 (3.1%)	5 (3%)	9 (3.3%)	3 (2.5%)
Ventricular arrhythmias *n* = 11 (3%)	6 (2.6%)	5 (3.2%)	4 (1.8%)	7 (4.2%)	7 (2.6%)	4 (3.4%)
Complete heart block *n* = 3 (1%)	1 (0.4%)	2 (1.3%)	1 (0.4%)	2 (1.2%)	1 (0.4%)	2 (1.7%)

All *p*-values were insignificant (Pearson’s chi-square test).

Fourteen patients (4%) died of a cardiovascular cause during the follow-up period. Ten of these patients (71%) had the metabolic syndrome according to the NCEP ATP III definition, compared with eight (57%) who satisfied the criteria based on the IDF definition; this difference was not statistically significant.

Seventy-six per cent of patients had a previous or a current history of smoking. Of these, 61% had the metabolic syndrome according to the NCEP ATP III criteria and 58% for the IDF criteria. No additional risk was noted in patients who had the metabolic syndrome, compared with those who did not.

In 240 patients, ejection fraction was assessed by echocardiography. Thirty-two per cent of these patients had abnormal left ventricular systolic function, with significantly more patients (69%, *p* = 0.04) in this group having the metabolic syndrome according to the NCEP ATP III definition, compared to those without. This difference was not significant for the IDF definition (*p* = 0.109). However, in patients with cardiac failure, 92% had elevated fasting blood glucose levels according to the IDF definition, compared to 56% for the NCEP ATP III definition.

## Angiographic studies

**Fig. 1.**
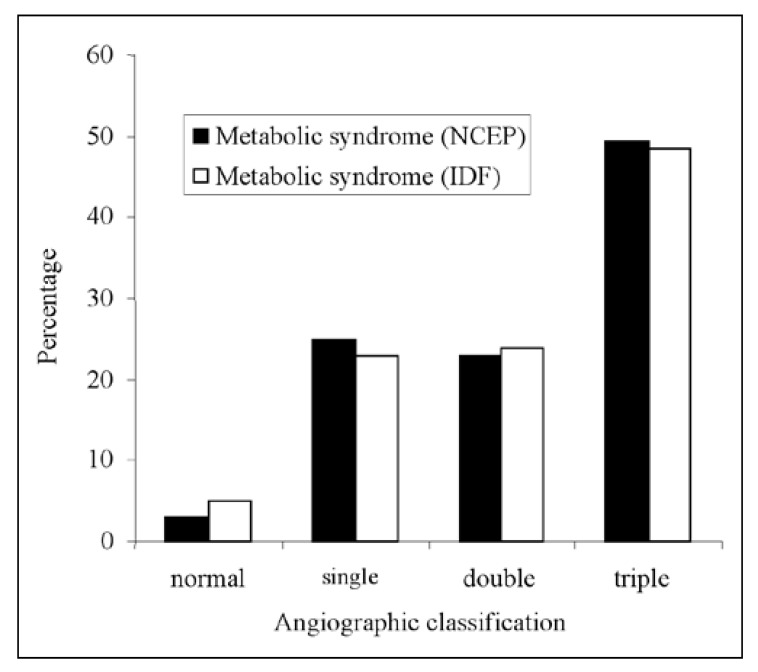
Angiographic classification of disease severity in relation to the metabolic syndrome.

Cardiac catheterisation studies were performed on 172 patients with 75 (44%) presenting with triple-vessel disease, 43 (25%) with double-vessel disease, 47 (27%) with single-vessel disease, while seven (4%) had normal coronary angio-grams. Overall, no significant difference in disease severity classified by angiography was seen between the two definitions (Fig. 1). Triple-vessel disease occurred commonly in patients with the metabolic syndrome (50 and 49% according to the NCEP ATP III and the IDF definitions, respectively). In patients who did not have the metabolic syndrome as defined by either criteria, only 34 and 37% (NCEP ATP III and IDF, respectively) had triple-vessel disease.

Thirty-one per cent of patients underwent coronary revascularisation, either by CABG (58%) or percutaneous coronary intervention (PCI) with or without stenting (42%). There were no significant differences in patients with or without the metabolic syndrome who were subjected to revascularisation procedures, for either the NCEP ATP III or IDF definition.

## Discussion

This study clearly demonstrates that irrespective of the type of definition used, there is a high prevalence of the metabolic syndrome in young South African Indian patients with premature MI. The similarity in the results obtained for the two definitions (60% for NCEP ATP III and 57% for IDF) was not unexpected. It has been demonstrated previously that prevalence figures for the metabolic syndrome are similar in any given population, regardless of the definition used, although different individuals may be identified within each group.^22^

What is of concern, however, is that there was only a 79% concordance between the two definitions (kappa = 0.565). Of the remaining 21% of patients with discordant results, 12% were diagnosed with the metabolic syndrome based on the NCEP ATP III definition only, while 9% had the syndrome solely according to the IDF definition.

Based on the suggested modifications to the NCEP ATP III definition by the AHA/NHLBI,^21^ which recommends using specific waist criteria for different ethnic groups as proposed by the IDF, we were able to diagnose significantly more patients with the metabolic syndrome compared with either of the accepted NCEP ATP III or IDF definitions (*p* < 0.001). This suggests that lower cut-off values for waist circumference should be used in populations that are at high risk of insulin resistance-mediated cardiometabolic disease, such as the Asian Indians.

Population studies have shown that the prevalence of the metabolic syndrome varies between different countries, particularly with respect to gender, age and ethnic origin.^22^ In studies that included subjects of all ages, the prevalence within urban populations in different countries varied from 8% (India) to 24% (USA) in men, and from 7% (France) to 43% (Iran) in women. The prevalence of the metabolic syndrome in young South African Asian Indian patients with AMI has not been reported on previously, and the high frequency of this syndrome observed here may contribute to the earlier onset of accelerated atherosclerotic disease in these subjects.

The distribution of body fat in Asian Indians also differs from that seen in Western populations, with Indians having a higher body fat content or abdominal obesity, even within normal ranges of body mass index.^23^ An increase in visceral fat is a reliable marker of insulin resistance and hyperinsulinaemia, which are risk factors for the development of hypertension, dyslipidaemia, diabetes and CHD.^24^ However, the application of lower ethnic-specific waist circumference cut-offs for obesity as proposed by the IDF had no significant impact on the prevalence of the metabolic syndrome in our patients. This raises some doubt, certainly in young Asian Indian patients, as to the validity of using central obesity as a prerequisite for the diagnosis of the metabolic syndrome.^25^ These findings suggest that obesity *per se* may not be the principal cause of the elevated cardiometabolic risk, but rather the cardiometabolic risk factors that are associated with obesity.

With respect to the various components of the metabolic syndrome, elevated fasting blood glucose was found to be the most frequently occurring factor in patients with the syndrome [IDF (91%), NCEP ATP III (86%)]. It is well documented that the metabolic syndrome is associated with an increased risk of diabetes^26^ and can predict the onset of future diabetes.^27^ In a study comparing Asian with European subjects, a higher prevalence of diabetes was found in the Asian subjects (19 vs 4%).^6^ Diabetes mellitus has also been shown to occur with a high frequency in South African Asian Indians,^3^ and this suggests that ethnic influences may be a contributory factor to the increased frequency of elevated fasting blood glucose in our patients with the metabolic syndrome. In addition, patients with the metabolic syndrome in our study also had significantly higher blood pressures, higher plasma triglycerides and lower HDL cholesterol concentrations compared to those without the syndrome (*p* < 0.001).

The metabolic syndrome has been associated with an increased incidence of cardiovascular disease and CHD mortality. 16 Our study supported this observation since patients with the metabolic syndrome according to the NCEP ATP III criteria were found to have significantly more severe left ventricular systolic dysfunction as assessed by echocardiography (*p* = 0.04), although this difference was not significant for the IDF definition. Furthermore, patients with the syndrome who were subjected to angiographic studies were found to have severe and diffuse premature atherosclerotic disease, with 50% having triple-vessel disease compared to 34% who did not have the syndrome. Similar results were observed when the IDF criteria were applied.

In a recent study on an unselected population of patients with AMI, the metabolic syndrome was shown to be associated with a worse in-hospital outcome, and a higher risk for the development of severe heart failure.^28^ In contrast, in the young patient population studied here, the occurrence of adverse cardiovascular events in hospital was relatively low, with recurrence of angina (9%) and cardiac failure (8%) being the most common adverse events. There were also no significant differences in adverse event rates in patients with and without the metabolic syndrome. Although the overall mortality rate was low in the study group (4%), patients with the syndrome, as defined by either definition, tended to have an increased risk of death compared to those without, but these differences were not significant.

Potential limitations of this study merit consideration. One such limitation was that the number of deaths and adverse cardiovascular events during hospitalisation were relatively small. Therefore, data regarding adverse events and mortality should be considered only suggestive. Furthermore, not every patient was assessed for left ventricular ejection fraction, and not all patients were subjected to cardiac catheterisation studies. Results on these smaller subgroups were therefore tentative. In addition, blood samples were taken in the fasting state during admission, when patients were acutely ill and this may have increased the number of patients with abnormal blood glucose and lipid values.

## Conclusion

The metabolic syndrome is a common finding in young Asian Indian patients with AMI. These patients present with extensive atherosclerotic disease on angiographic studies, but this does not appear to adversely affect the in-hospital complication rate and short-term outcome. There was no significant difference in the prevalence rate of the metabolic syndrome as determined by either the NCEP ATP III or IDF definitions but there was only a moderate level of agreement between the two definitions.

As suggested by the current study, inclusion of ethnic-specific waist circumference cut-offs as the criterion for obesity in the NCEP ATP III definition may assist in the diagnosis of the metabolic syndrome, and more accurately identify individuals at risk. Clearly larger studies are required to confirm these findings.
